# Molecular Composition of Heterochromatin and Its Contribution to Chromosome Variation in the *Microtus thomasi*/*Microtus atticus* Species Complex

**DOI:** 10.3390/genes12060807

**Published:** 2021-05-25

**Authors:** Michail Rovatsos, Juan Alberto Marchal, Eva Giagia-Athanasopoulou, Antonio Sánchez

**Affiliations:** 1 Department of Ecology, Faculty of Science, Charles University, 12800 Prague, Czech Republic; michail.rovatsos@natur.cuni.cz; 2 Department of Experimental Biology, Faculty of Experimental Sciences, University of Jaén, Campus Las Lagunillas s/n, E-23071 Jaén, Spain; jamaor@ujaen.es; 3 Section of Animal Biology, Department of Biology, University of Patras, GR-26500 Patras, Greece; giagia@upatras.gr

**Keywords:** *Microtus*, Msat-160, polymorphism, repeated DNAs, sex chromosomes heterochromatin, telomeric sequences

## Abstract

The voles of the *Microtus thomasi*/*M. atticus* species complex demonstrate a remarkable variability in diploid chromosomal number (2n = 38–44 chromosomes) and sex chromosome morphology. In the current study, we examined by in situ hybridization the topology of four satellite DNA motifs (Msat-160, Mth-Alu900, Mth-Alu2.2, TTAGGG telomeric sequences) and two transposons (LINE, SINE) on the karyotypes of nine chromosome races (i.e., populations with unique cytogenetic traits) of *Microtus thomasi*, and two chromosomal races of *M. atticus*. According to the topology of the repetitive DNA motifs, we were able to identify six types of biarmed chromosomes formed from either Robertsonian or/and tandem fusions. In addition, we identified 14 X chromosome variants and 12 Y chromosome variants, and we were able to reconstruct their evolutionary relations, caused mainly by distinct mechanisms of amplification of repetitive DNA elements, including the telomeric sequences. Our study used the model of the *Microtus thomasi*/*M. atticus* species complex to explore how repetitive centromeric content can alter from chromosomal rearrangements and can shape the morphology of sex chromosomes, resulting in extensive inter-species cytogenetic variability.

## 1. Introduction

Sex chromosomes evolved several times independently in different lineages. The evolutionary dynamics of the X and Y chromosomes are defined by specific features in terms of gene composition as well as in evolutionary rates. Thus, X chromosome gene content and order are extremely conserved between distantly related species of mammals [1], with only minor exceptions described in some rodents [2]. By contrast, the Y chromosome has been losing most of its original gene content over evolutionary time, a well-known process named “Y chromosome degeneration” [3]. This explains the enormous variation in size, gene content, and structural complexity observed even in closed related species [4]. Usually, the Y chromosome is small, nearly full heterochromatic, and almost depleted of genes in the majority of mammal species.

The subfamily Arvicolinae comprises a mammalian group of special interest for studies on karyotype and sex chromosome evolution. It includes more than 150 extant species established during recent and rapid radiation events, which resulted in high karyotypic variation [5]. Karyotype evolution encompassed a series of fusion/fission rearrangements, inversions, and centromeric shifts, accompanied by other cryptic intrachromosomal rearrangements [6,7], leading to extensive variability in chromosome numbers ranging from 2n = 17 in *Ellobius lutescens* to 2n = 62 in *M. duodecimcostatus* and *M. lusitanicus* [8,9].

Some species from the genus *Microtus* are characterized by atypical sex-related genetic features that range from an unusual sex determination system to extensive sex chromosome polymorphisms [10,11,12,13,14,15,16,17,18,19,20,21,22,23,24]. In the genus *Microtus*, a numerous collection of variants evolved from an ancestral sex chromosome pair, even within related species or at intraspecific level [8,25,26,27,28]. The evolution of X and Y chromosomes in the voles of the genus *Microtus* occurred very fast and was the result of intrachromosomal rearrangements and amplification of repeated DNA sequences [29,30]. In some species (*M. agrestis*, *M. cabrerae*, *M. chrotorrhinus*, *M. rossiaemeridionalis*, and *M. transcaspicus*), these processes resulted in enlarged (giant) sex chromosomes containing large heterochromatic blocks [16,31]. While the size and content of the euchromatin are highly conserved in the giant sex chromosomes, their heterochromatic composition differs abruptly at both intraspecific and interspecific levels [11,15,16,17,32,33,34,35].

One of the most remarkable examples of chromosome polymorphism is present in the sibling species of the subgenus *Terricola*; *Microtus thomasi* (2n = 38–44) and *M. atticus* (2n = 44). The two closely related species demonstrate extensive chromosomal polymorphism, resulting in the descriptions of nine chromosomal races (i.e., geographical isolated populations with specific chromosome constitution, defined by the presence of chromosomal rearrangements, mainly Robertsonian and tandem fusions) in *M. thomasi* and two chromosomal races in *M. atticus* (see Table 1) [23,36,37,38,39,40,41,42,43,44]. Although the chromosomal races correspond to parapatric populations, hybrids between voles from different chromosomal races of *M. thomasi* can occur in the population contact zones (Table 1). Beyond the polymorphism on chromosome morphology, resulting in the description of the above chromosomal races, additional polymorphism on morphology, size, and repetitive content was revealed on the sex chromosomes (see Table 1) [36,37,38,39,40,41,42,43,44]. The combination of C-banding and sex chromosome painting data have redefined the original polymorphism established on the basis of morphological features [23]. That is, at least 14 X chromosome and 12 Y chromosome variants are recognized today in wild populations. X chromosomes with either acrocentric (X_1_ to X_12_) or submetacentric (X_13_ to X_14_) morphology and with variable amounts of heterochromatin were described. The Y chromosome variants are either acrocentric (Y_1_ to Y_9_) or metacentric (Y_10_ and two Y_12_), most of them being fully heterochromatic but differing in size [23,36,37,38,39]. Four different types of repeated DNAs have been characterized from the pericentromeric heterochromatin of these species: Msat-160 [42], Mth-Alu900 [36], Mth-Alu2.2 [39], and telomeric-like sequences [43]. However, the detailed contribution of those repeated sequences to the molecular differentiation of the karyotype variants of these species remains unknown, as only partial data were previously published [36,39,43].

In the current study, we aspire to explore the contribution of the repetitive elements (e.g., satellite motifs and transposons) in the formation and polymorphism of the heterochromatic blocks of the sex chromosomes, using as a model the *M. thomasi*/*M. atticus* species complex, a group of voles with extensive polymorphism of sex chromosomes (morphology, heterochromatin, and repetitive motif content). Therefore, we have performed a detailed comparison of the topology of four satellite DNA repeats (Msat-160, Mth-Alu900, Mth-Alu2.2, and TTAGGG telomeric sequences) and two transposons (LINE, SINE) on ten chromosome races of *M. thomasi*, and two chromosomal races of *M. atticus*. From our comparative cytogenetic analysis, we tried to reconstruct that the putative events and evolutionary paths underlining the appearance of the biarmed autosomes and the sex chromosome variants. Moreover, we identified the repeated DNA motifs involved in the heterochromatin enlargement processes experienced by the X and Y chromosome variants, a hallmark of sex chromosome evolution in *Microtus* rodents.

## 2. Materials and Methods

### 2.1. Individuals Analysed and Chromosome Preparations

**Figure 1:**
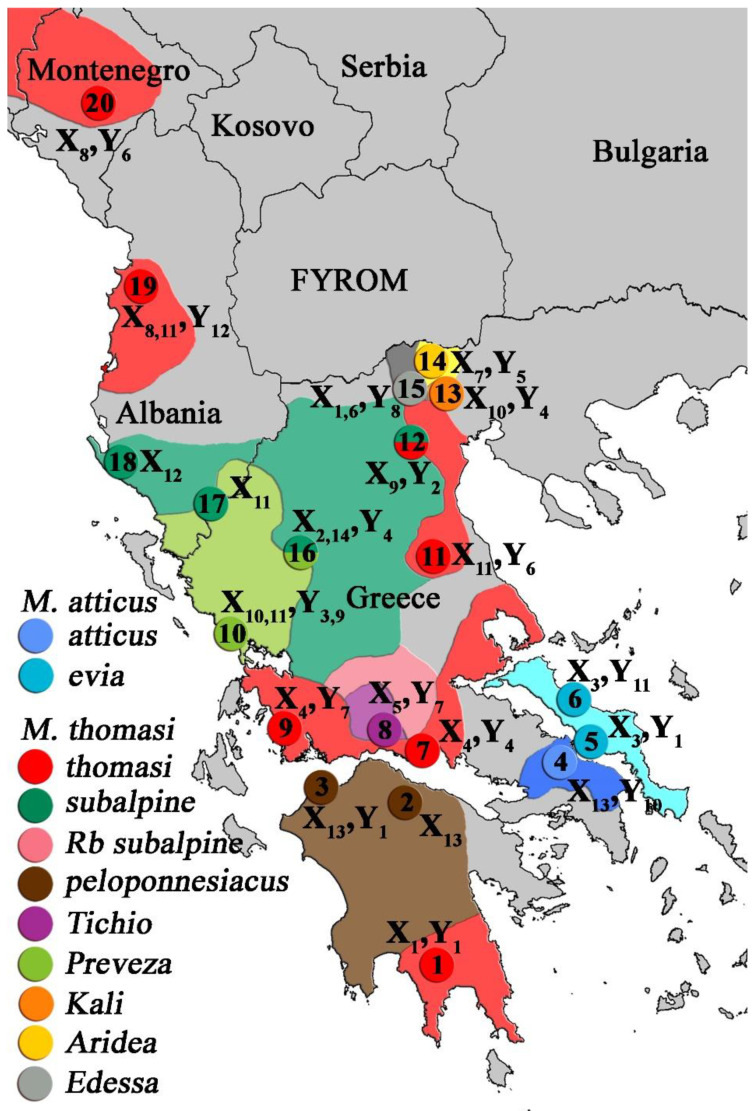
Distribution of the chromosomal races of the species *M. thomasi* and *M. atticus*. Sex chromosomes variants and sampling points are indicated: 1: Xirokampi; 2: Kalavryta; 3: Strofylia; 4: Afidnes; 5: Chalkida; 6: Kimassi; 7: Galaxidi; 8: Ano Tichio; 9: Astakos; 10: Kastrosikia; 11: Tyrnavos; 12: Veria; 13: Kali; 14: Aridea; 15: Edessa; 16: Mpalntouma; 17: Kakavia; 18: Ducat; 19: Preze; 20: Donje Selo.

A detailed description of the vole specimens from Greece, Albania, and Montenegro utilized in this work is included in Rovatsos et al. [23,39]. We used chromosome preparations from 26 individuals of different chromosomal races of the two species from the subgenus *Terricola* of the genus *Microtus: M. thomasi* and *M. atticus* (Table 1; Figure 1). Chromosome preparations were obtained from bone marrow using a modified version of the Hsu and Patton [45] method. The karyotypes of the analyzed individuals have different sex chromosome constitutions, which were recognized by their C-banding and chromosome painting profiles [23,37,38].

### 2.2. Probes and FISH

We used for FISH six probes of different repeated sequences; the satellite DNA Msat-160 characterized in several *Microtus* species [42], the Mth-Alu900 and Mth-Alu2.2 repeats, both characterized in *M. thomasi* and *M. atticus* [36,39], the telomeric sequences TTAGGG [43], one LINE1 fragment, and one SINE sequence. LINE and SINE probes were derived from the analyses of clones containing a collection of repeated DNA sequences isolated from the *M. atticus* genome by *Alu*I restriction [36,39]. In detail, for the LINE1 probe, a clone was used containing a fragment of the OFR2 of this retroelement (854) (GenBank accession number: MW767177), while the SINE probe (150 bp) was obtained via PCR with specific primer pair on a clone containing a sequence related to mouse B1/primate Alu element within its insert. (GenBank accession number; MW767176). Biotin labeling of probes and FISH was performed as described by Rovatsos et al. [43].

## 3. Results

### 3.1. Hybridization Profile of Repetitive DNA Motifs in Autosomes

**Figure 2:**
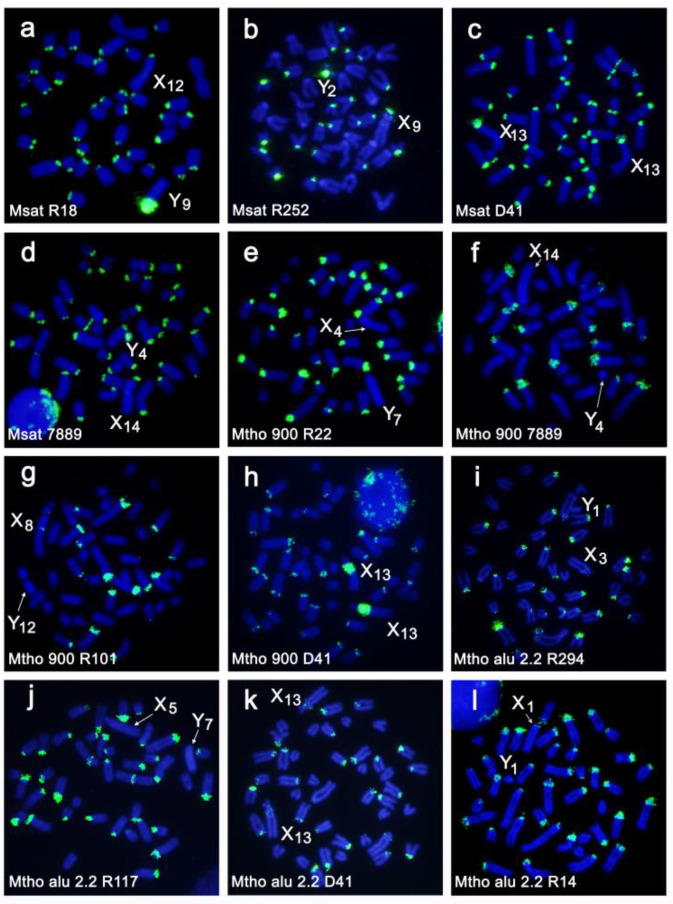
Representative examples of FISH with the probes Msat-160 (**a**–**d**), Mth-Alu900 (**e**–**h**), and Mth-Alu2.2 (**i**–**l**) on chromosomes of different individuals. The individual identification and sex chromosome variants are indicated.

**Figure 3:**
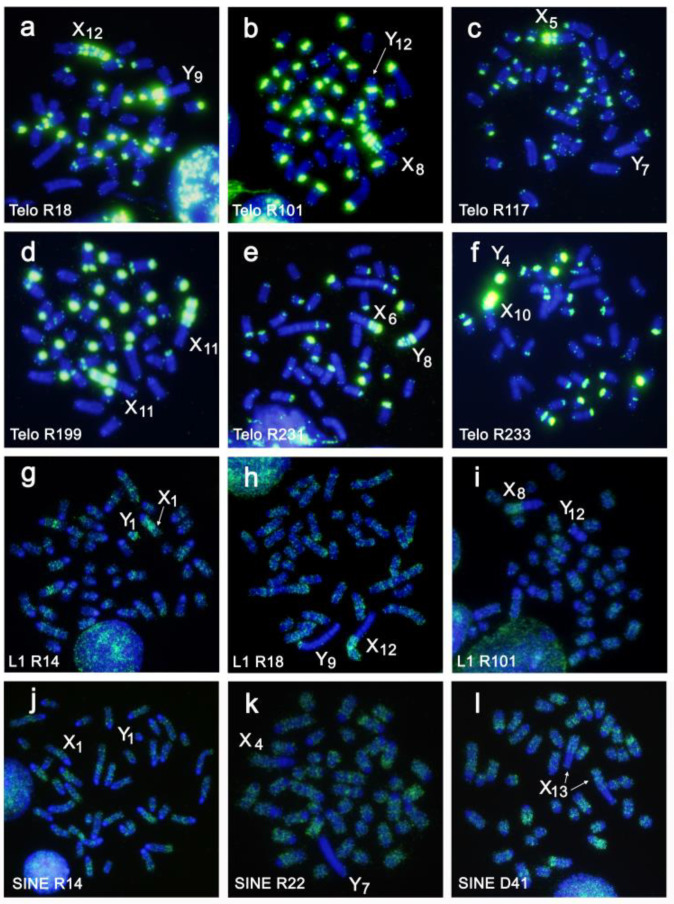
Representative examples of FISH with the probes of telomeric sequences (**a**–**f**), LINE1 (**g**–**i**), and SINE (**j**–**l**) on chromosomes of different individuals. The individual identification and sex chromosome variants are indicated.

**Figure 4:**
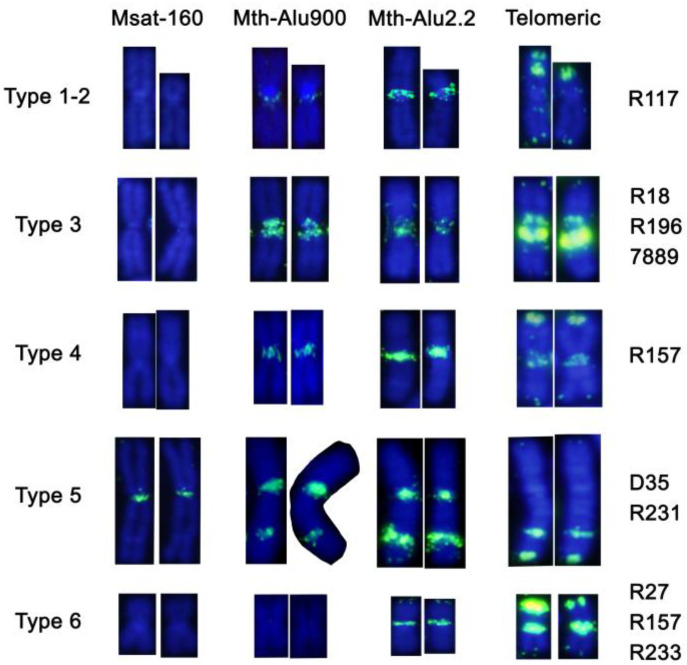
FISH pattern of the biarmed autosomes hybridized with Msat-160, Mth-Alu900, Mth-Alu2.2, and telomeric sequences. The codes of the individuals (see Table 1) with these chromosomes in their karyotype are indicated on the right.

Chromosome numbers in the analyzed specimens ranged from 2n = 38 to 2n = 44 due to variation in the number of autosomes, with the exception of specimen R32 containing 45 chromosomes due to an XXY sex chromosomes constitution (Rovatsos et al. 2008). Most of the autosomes were acrocentric; however, biarmed chromosomes were present in nine specimens, probably as a consequence of Robertsonian and tandem fusion rearrangements [40,41]. The repeated sequences-Msat-160, Mth-Alu900, Mth-Alu2.2, and telomeric sequences-were enriched in the pericentromeric heterochromatin of most autosomes, although variation in the number of hybridized chromosomes and in the intensity of the signals was observed (Figure 2a–l and Figure 3a–f). Based on the morphology and the detailed analyses of the FISH signals produced by these four repetitive sequence probes, we could distinguish six different types of biarmed chromosomes; biarmed type 1–6 (Figure 4).

Biarmed types 1 (metacentric) and 2 (submetacentric) were both enriched for Mth-Alu2.2 repeats in the pericentromeric heterochromatin (Figure 4). Moreover, in type 1, telomeric repeats accumulated as two intense bands in the p-arm and two faint interstitial bands in both arms. The terminal band enriched in telomeric repeats from type 1 was missing in type 2 (Figure 4). Both types lacked Msat-160 satellite, and Mth-Alu900 only produced a very faint signal in their pericentromeric regions. Biarmed type 1 and 2 were present only on the specimen (R117), a hybrid between the chromosomal races Tichio and Rb-subalpine. Type 1 was the Rb metacentric of the Rb-subalpine race, and type 2 was the submetacentric chromosome of the Tichio race.

Biarmed type 3 included two bands of Mth-Alu900 and Mth-Alu2.2 repeats the pericentromeric regions of both arms. Moreover, telomeric repeats accumulated as bright interstitial bands surrounding the centromere. Msat-160 repeats were not observed (Figure 4). Type 3 chromosome was present in three specimens (7889, R196, and R18) and characterized the Preveza race.

Biarmed type 4 showed Mth-Alu900, Mth-Alu2.2, and telomeric repeats signals in the centromeric region. In this case, telomeric repeats were also heavily enriched at the terminal position of the p-arm. Msat-160 repeats were not included (Figure 4). This type was only present in one specimen of the Aridea race (R157).

Biarmed type 5 was characterized by enrichment of Msat-160, Mth-Alu900, and Mth-Au2.2 repeats in the pericentromeric region. Interestingly, it also showed an interstitial band in the middle of the q-arm positive for Mth-Alu900, Mth-Alu2.2, and telomeric sequences. Telomeric repeats are also highly accumulated at the terminal position of the q-arm (Figure 4). This type defines Edessa race and is present in two specimens (231 and D35) at homozygous and hemizygous status, respectively.

Biarmed type 6 showed enrichment of Mth-Alu2.2 and telomeric repeats in its pericentromeric region. Telomeric repeats also accumulated at the terminal p-arm producing a bright, intense band. Neither Msat-160 nor Mth-Alu900 sequences were detected (Figure 4). This type was typical of Kali and Aridea races and was identified in three specimens (R233, R27, R157). Interestingly, specimen 157 included biarmed type 4 and type 6.

### 3.2. Hybridization Profile of Repetitive DNA Motifs in Sex Chromosome Variants

**Figure 5:**
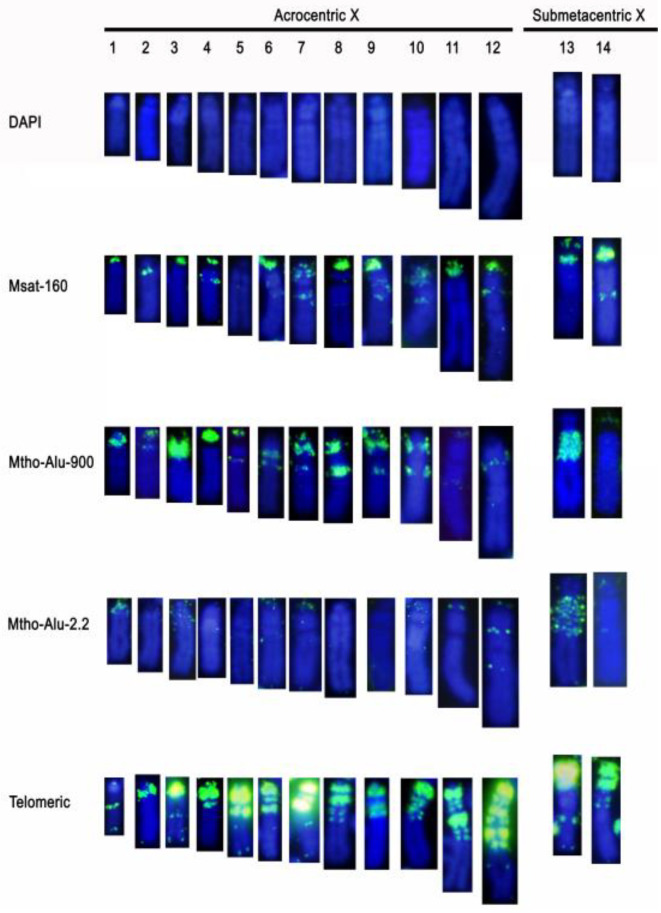
X chromosome variants showing the FISH pattern with the probes Msat-160, Mth-Alu900, Mth-Alu2.2, and telomeric sequences, and DAPI staining.

**Figure 6:**
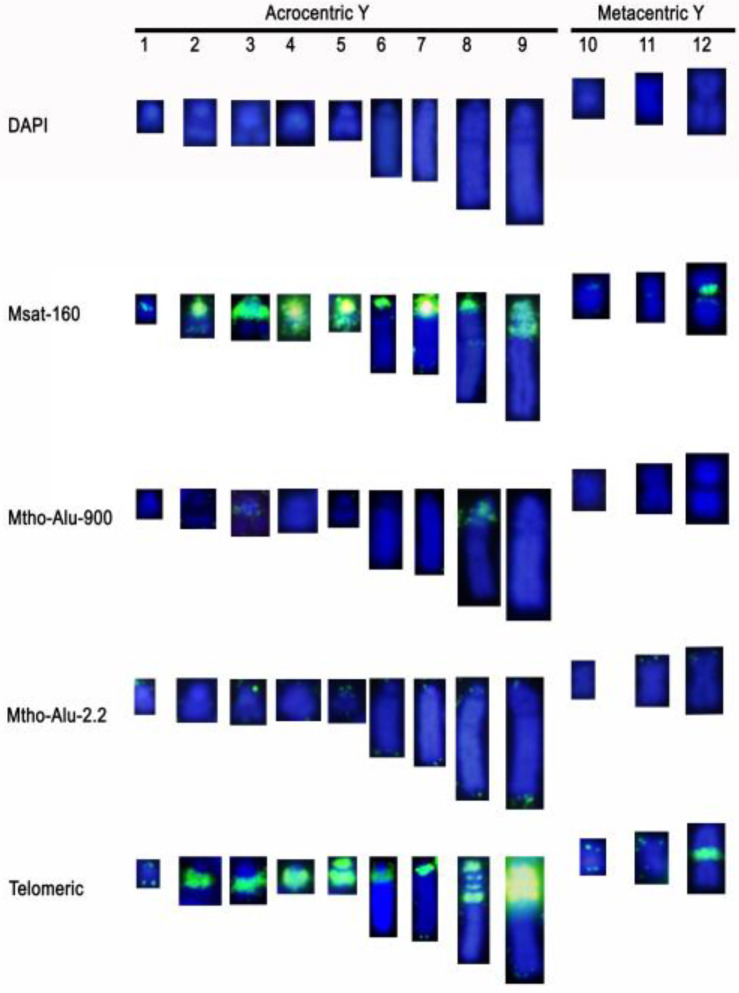
Y chromosome variants showing the FISH pattern with the probes Msat-160, Mth-Alu900, Mth-Alu2.2, and telomeric sequences, and DAPI staining.

Msat-160 sequences were present in all X chromosome variants, with the exception of chromosome X_5_. This probe produced a signal in one pericentromeric band and/or in a more interstitial band in some of them. The submetacentric X variants showed differences: X_13_ had one terminal band in the p-arm and another in the pericentromeric heterochromatin, while X_14_ had a signal in an interstitial band in the q-arm and in almost the entire short arm (Figure 5). Concerning the Y chromosome, Msat-160 was enriched in the pericentromeric heterochromatin of all acrocentric variants and from the biggest metacentric (Y_12_). In Y_2_, Y_4_, and Y_5_, Msat-160 was also distributed in variable intensity along the entire chromosome. In Y_9_ this repeat sequence is organized in three-wide bands closed to the centromeric region. In the metacentric, Y_10_ Msat-160 was restricted only at the subtelomeric position, while in the metacentric Y_11_, the signal was nearly absent (Figure 6). Mth-Alu900 repeats show a distribution similar to Msat-160 within the X chromosome variants. Thus, they are included in one pericentromeric band and, in some cases, in a more interstitial band (Figure 5). In relation to the Y variants, only the acrocentric Y_3_ and Y_8_ contain this repeated DNA sequence in their pericentromeric regions (Figure 6).

Mth-Alu2.2 produced a very weak signal in the pericentromeric heterochromatin of the acrocentric X variants, which was difficult to detect in some cases. Some of them showed additional interstitial bands of faint nature. Only X_3_ had a clear dot signal along the heterochromatic block of the q-arm. The submetacentric X_13_ had hybridization signals on the heterochromatic block of the q-arm, while X_14_ contained only a fine terminal band on the p-arm (Figure 5). Like the X chromosomes, most acrocentric Y chromosomes presented very weak signals of this repeat on pericentromeric heterochromatin and in one telomeric region of the larger acrocentrics and metacentrics (Figure 6).

Telomeric repeats were heavily enriched in the heterochromatic regions of most acrocentric X variants, while X_1_ did not contain them (Figure 5). The banding pattern revealed from C-banding and telomere-FISH indicates that in several cases, the X chromosome variants might have originated by subsequent duplications of heterochromatic blocks, such as in the variants X_10_, X_11_, and X_12_, where a heterochromatic block consisting of a thick band followed by two smaller bands, all three enriched in telomeric-like sequences seemed to form a single, double, and triple blocks in each of these variants (Figure 5). Both submetacentric X chromosomes contained abundant telomeric sequences along the p-arm and in one band closed to the pericentromeric region of the q-arm. Additionally, X_14_ had two interstitial bands within the heterochromatic block (Figure 5).

In the acrocentric Y-chromosome, telomeric repeats accumulated in the pericentromeric heterochromatin and the nearest regions, with the exception of Y_1_. In the smaller variants, the signal occupied almost all of the long arm; in Y_5_, it was enriched in the minute small arm, while in the larger variants (Y_8_ and Y_9_), it was observed a banding pattern, which resembled that observed in the X chromosomes. From the metacentric Y chromosomes, only Y_12_ showed a very strong pericentromeric heterochromatin signal (Figure 6).

Finally, we also analyzed the hybridization pattern of LINE1 and SINE sequences in these species. Our data clearly show, as was previously demonstrated in voles [46,47], that both retroelements are interspersed along the euchromatic regions of all chromosomes in a variable manner. As expected, LINE1 sequences were enriched in the euchromatin of all Xs variants. However, both sequences were depleted from the heterochromatic regions of the X and Y variants investigated (Figure 3g–l).

## 4. Discussion

### 4.1. Autosomal Karyotype Variation

In voles there are some examples of species with remarkable intraspecific variation in their diploid chromosome number, e.g., *M. logicaudus* (2n = 56 to 2n = 70) [48], and *M. maximowiczii* (2n = 36 to 2n = 44) [49]. Our study is focused in two sibling species, *M. thomasi* and *M. atticus*, showing variation in diploid chromosome numbers (2n = 38 to 44), fundamental numbers (FN = 40 to 46) and sex chromosome constitution [23,38,40,41,50,51,52]. Based on those karyotype variations, several parapatric chromosomal races along Greece, Albania, and Montenegro were established [23,38,40]. The karyotype of the chromosomal race assigned as *M. thomasi ‘thomasi’* with all chromosomes being acrocentric (2n = 44, FN = 44) was considered the most ancestral, from which the other chromosomal races evolved, including *M. atticus* variants [52]. Although hybrids between some races were described in the wild [37,39], *M. thomasi* races are well differentiated and probably reproductive isolated from the two *M. atticus* races, as concluded from cytochrome b gene analysis and reproductively successful assays [40].

Karyotype polymorphism within these chromosomal races could be partially explained by the variable presence of biarmed chromosomes [40,41]. Our study identified different types of sex chromosomes based on the distribution of satellite motifs and repetitive elements in the heterochromatic regions. The distribution of the investigated repeated sequences suggests that they probably evolved from different acrocentric pairs. Our preliminary data from comparative chromosome painting seem to confirm this hypothesis. Biarmed types 2, 3, 4, and 6, which defined Tichio, Preveza, Aridea, and Kali chromosomal races, respectively, could have originated from Robertsonian fusion of acrocentric chromosomes depleted of Msat-160 sequences at their centromeres. The acrocentric chromosomes involved in type 1 and type 6 formation were also lacking telomeric and Mth-Alu900 pericentromeric sequences, respectively. Alternatively, those sequences could be lost during the rearrangement processes. Type 1, which defines the chromosomal race Rb-subalpine, arose probably from type 2 upon a tandem fusion with one small acrocentric chromosome. Types 2, 4, and 6 all next achieved amplification of telomeric repeats in the terminal region of the p-arm. Finally, type 5 defined the Edessa race and could have evolved after the fusion of two acrocentric chromosomes depleted of telomere repeats at their centromeres. Afterward, a tandem fusion with a small acrocentric chromosome could explain the increased length of the q-arm and the occurrence of one interstitial band composed of repeats.

Vole karyotypes are prone to chromosomal rearrangements, most commonly Robertsonian and tandem fusions, as well as pericentric and paracentric inversions [27,53,54]. An observable trend of karyotypic evolution in voles is the progressive reduction in chromosomal number due to chromosome fusions [53]. Our results show that pericentromeric heterochromatin of *M. thomasi* and *M. atticus* acrocentric chromosomes was highly complex and variable, with up to four different families of repeated DNA included. The occurrence of Robertsonian and tandem fusions could have been favored in acrocentric chromosomes containing a highly dynamic and enlarged centromeric heterochromatin. Further fixation of the new chromosome variants due to inbreeding and random genetic drift in rather isolated sub-populations could explain the extensive chromosome variation observed in these two species [23,37,38,55].

Meiotic drive, the process altering the probability for a chromosome to segregate to ovum or to the polar body during female gametogenesis, might be involved in the formation of chromosomal races in the *M. thomasi/M. atticus* complex. The meiotic drive seems to act based on the affinity of spindle microtubules to centromeres [56,57,58], and it is considered a protective mechanism to filter chromosomal aberrations which alter the centromere structure, such as Rb-fusions, by driving them to the polar body. Although this scenario might occur, e.g., in the case of *Mus muscuslus domesticus* [59], it is possible that certain Rb-fusions might not alter the centromere structure, and therefore, remain hidden from the control of the meiotic drive. We speculate that the bi-armed chromosomes of *M. thomasi* (Figure 4; types 1–6) bypassed the control of the meiotic drive because they maintained an almost intact satellite content (Mth-Alu900, Mth-Alu2.2, and the telomeric-like repeats) in the centromeric regions after the Rb-fusion. One future direction of research could be to test if the novel centromeres of these Rb-chromosomes could be strong meiotic drivers, which could lead to their fast spreading in the population and could explain the formation of the numerous parapatric chromosomal races in the *M. thomasi/M. atticus* complex.

### 4.2. Sex Chromosomes Variation

**Figure 7:**
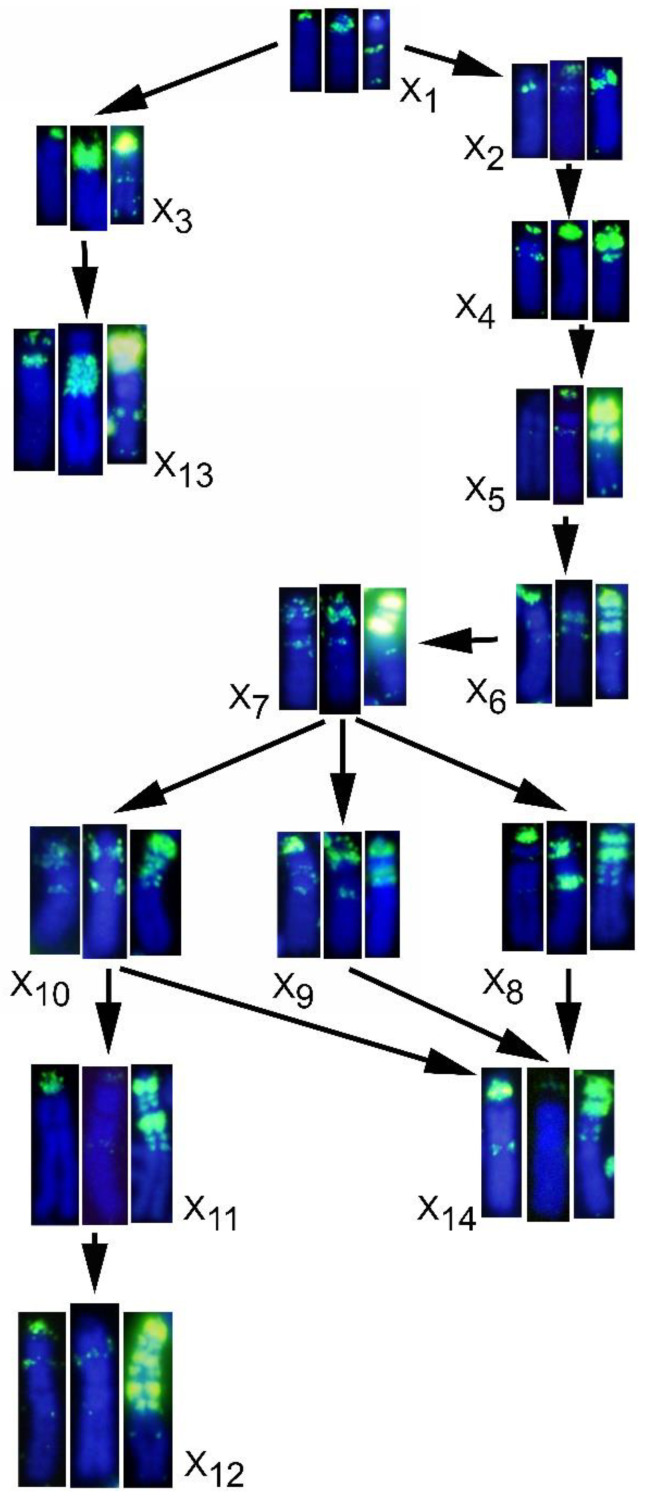
Most plausible evolutionary paths of the X chromosome variants. From left to right, chromosomes hybridized with Mast-160, Mth-Alu 900 and telomeric sequences.

**Figure 8:**
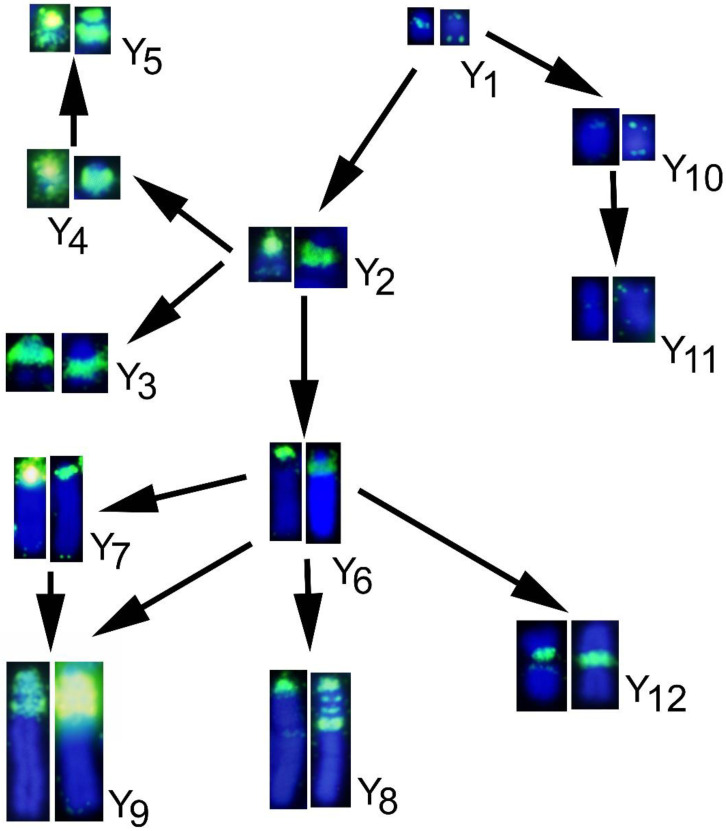
Most plausible evolutionary paths of the Y chromosome variants. From left to right, chromosomes hybridized with Mast-160, and telomeric sequences.

Painting with different sex chromosome probes demonstrated the existence of at least fourteen X chromosome and twelve Y chromosome variants in *M. thomasi* and *M. atticus* [23]. Twelve X chromosomes (X_1_ to X_12_) are acrocentric and two (X_13_ to X_14_) submetacentric, while five Y chromosomes were acrocentric of small size (Y_1_ to Y_5_), four acrocentric large (Y_6_–Y_9_), and three metacentric chromosomes of variable size (Y_10_–Y_12_) (Figure 5, Figure 6, Figure 7 and Figure 8). All of these variants evolved most probably from the smallest ones (X_1_ and Y_1_), considered to be the ancestral sex chromosomes of this species. Our present study unraveled the full picture about the dynamics of different types of repeated DNAs within all these sex chromosome variants. The heterochromatin enlargement in the sex chromosomes probably involved gradual amplification of repetitive DNA sequences during the northward colonization of the species from a southern glacial refuge [37,38]. Based on a previous chromosome painting study of the geographical distribution of sex chromosome types [23] and our new data, we propose evolutionary paths explaining the formation of X and Y chromosome variants.

We could distinguish two main lineages in the X chromosomes (Figure 7). We assume that the X_1_ type is the ancestral one for both *M. thomasi* and *M. atticus*. In the first lineage of X types (X_3_, X_13_), an ancestral X_1_ possibly gave rise to X_3_ through extensive enrichment of telomeric Mth-Alu 900 repeats in the heterochromatic regions and with minor involvement of Msat-160 and Mth-Alu2.2 repeats. Further amplification of these sequences, together with enrichment of telomeric repeats in the p-arm could have led to the X_13_ type (Figure 7). Both X_3_ and X_13_ exist in *M. atticus*, while the X_13_ also exists in *M. thomasi*.

In the second lineage of X types (X_2_, X_4_–X_12_, and X_14_), we propose successive rounds of amplification of Msat-160, Mth-Alu900, and telomere repeats, combined with some reduction episodes, gradually transformed the X_1_ ancestral type into types X_2_–X_7_. Some tandem duplication of heterochromatic bands and paracentromeric inversions must also have been involved. Similar processes might account for the evolution of the other variants from X_7_. By consecutive duplication events of heterochromatin, X_11_ and X_12_ could have evolved gradually from X_10_. The evolution of X_14_ could occur either from X_8_, X_9_, or X_10_; in this case, the short arm evolved by telomeric enlargement in an originally minute small arm, or alternatively by a pericentric inversion. It is noteworthy that X_13_ and X_14_ were found to be highly evolutionary distant despite their similar morphology (Figure 7).

Concerning the Y chromosome, three main lineages might be defined, starting from Y_1_. We assume that the Y_1_ type, which is commonly shared in both *M. thomasi* and *M. atticus*, was probably existing in their most common ancestor. The first lineage of Y chromosomes (Y_10_ and Y_11_), which were present exclusively in *M. atticus*, a pericentric inversion in the Y_1_ could have given rise to Y_10_, and further heterochromatin amplification could have produced the Y_11_ type. None of the four satellite motifs tested in this study hybridized in the heterochromatic regions of the Y_10_ and Y_11_ types, and therefore, we assume that the formation of these types probably involved other, currently unknown repetitive elements.

The second lineage of Y chromosomes (Y_2_–Y_5_), presented exclusively in *M. thomasi*, the Y_2_ type evolved from the Y_1_ type, by amplification of Msat-160, telomeric repeats, and other unknown sequences, since a considerable part of the Y_2_ type is not covered by hybridization signal from the tested satellite motifs. Additional rounds of heterochromatin amplification combined with para- and pericentric inversions might explain the occurrence of Y_3_, Y_4_, and Y_5_ (Figure 8).

The third lineage of Y chromosomes (Y_6_–Y_9_, and Y_12_), present exclusively in *M. thomasi*, might have evolved from Y_2_ type by the addition of a large heterochromatic block composed mainly of unknown sequences leading to Y_6_ type. One pericentric inversion on Y_6_ could have produced Y_7_. Further duplications of the Msat-160- enriched pericentromeric heterochromatin and telomere repeats of either of these two variants might explain the evolution of the Y9 type. Y8 could have originated similarly but without the enlargement of Msat-160 regions. Finally, a pericentric inversion could have produced Y12 from Y6 (Figure 8).

Interspecies chromosome polymorphism, leading to the description of geographical isolate populations with unique cytogenetic traits (e.g., description of chromosome races based on the occurrence of unique Rb-fusions), is not uncommon in mammals, with the house *Mus domesticus* and the common shrew *Sorex araneus* consisting the most prominent examples [60,61,62]. On the contrary, extensive polymorphism on the morphology and heterochromatic content of sex chromosomes is rather uncommon in mammals, with the voles of the *M. thomasi*/*M. atticus* species complex being rather exceptional in this respect.

Taken together, our data highlight the complexity and variability in terms of sequences and structure of the repeated sequences arranged at the X and Y variants of these species. It is tentative to speculate that the burst of sex chromosome variants of *M. thomasi/atticus* represents the initial evolutive scenario towards giant-sized X and Y chromosomes experienced by other related species of this genus.

## 5. Conclusions

Our study examined the detailed contribution of four satellite DNA motifs (Msat-160, Mth-Alu900, Mth-Alu2.2, and TTAGGG telomeric sequences) and two transposons on the molecular differentiation of the karyotype variants of *Microtus thomasi*/*atticus* species complex. The variable presence of biarmed autosomes is mainly explaining variations in autosomal numbers within the chromosomal races. From our analyses, six different types of biarmed chromosomes were identified based on the composition of their heterochromatic regions. Most probably, these biarmed chromosomes evolved from different autosomal pairs.

We also demonstrated the existence of a complex pattern for the heterochromatic regions included in the X and Y variants. From the investigated sequences, three satellites—Mast-160, Mth-Alu900, and telomeric motifs—were mainly involved in the heterochromatin enlargement process at the X chromosome. For the Y chromosome, Msat-160 and telomeric motifs were the major drivers of heterochromatin amplification in this chromosome. It should be remarked that some X and Y variants still contain large stretches of heterochromatin composed of other unknown repeats. Moreover, our data demonstrate that the repetitive DNAs content and its organization pattern are shared to some extent between the heterochromatic regions of the X and Y chromosomes, in line with previous evidence [23].

In conclusion, *Microtus thomasi/atticus* species genomes include highly dynamic and complex heterochromatin. The occurrence of such complex dynamic heterochromatin is considered a hallmark of *Microtus* genomes [38] which might explain in part the occurrence of unstable rearranged karyotypes in these species.

## Figures and Tables

**Table 1:** Specimens of *M. thomasi* and *M. atticus* analyzed with an indication of the locality of trapping, the specimen code, the chromosomal race, the chromosome number (2n), the fundamental number (FN), sex, and the sex chromosomes variants (pX-chr, pY-chr).

Locality	Specimen	*Chr. Race* ^a^	2n	FN	Sex	pX-chr ^b^	pY-chr ^b^
*M. thomasi*							
Xirokampi, Greece	R14	“thomasi”	44	44	male	X_1_	Y_1_
Kalavryta, Greece	D41	“peloponnesiacus”	44	46	female	X_13_	
Strofylia, Greece	R32 *	“peloponnesiacus”	45	47	male	X_13_	Y_1_
Galaxidi, Greece	R33	“thomasi”	44	44	male	X_4_	Y_4_
Ano Tichio, Greece	R117	hybrid “tichio” × “Rb subalpine”	41	43	male	X_5_	Y_7_
Astakos, Greece	R22	hybrid “thomasi” × “Rb subalpine”	44	44	male	X_4_	Y_7_
Mpalntouma, Greece	D20	hybrid “thomasi” × “Rb subalpine”	43	44	female	X_14_–X_2_	
Mpalntouma, Greece	7889	hybrid “Preveza” × “Rb subalpine”	41	44	male	X_14_	Y_4_
Kastrosikia, Greece	R196	“Preveza”	40	42	male	X_11_	Y_3_
	R18	“Preveza”	40	42	male	X_12_	Y_9_
Tyrnavos, Greece	R30	“thomasi”	44	44	male	X_11_	Y_6_
Kali, Greece	R233	“Kali”	40	42	male	X_10_	Y_4_
Kali, Greece	R27	“Kali”	40	42	female	X_10_–X_10_	
Aridea, Greece	R157	“Aridea”	38	42	male	X_7_	Y_5_
Aridea, Greece	R152	“Aridea”	38	42	male	X_7_	Y_5_
Edessa, Greece	R231	“Edessa”	38	40	male	X_6_	Y_8_
	D35	hybrid “Edessa” × “thomasi”	41	42	male	X_1_	Y_8_
Kakavia, Greece	R199	“subalpine”	42	42	female	X_11_–X_11_	
Veria, Greece	R252	“subalpine”	42	42	male	X_9_	Y_2_
Ducat, Albania	R160	“subalpine”	42	42	female	X_12_–X_12_	
Preze, Albania	R94	“thomasi”	44	44	male	X_11_	Y_12_
	R101	“thomasi”	44	44	male	X_8_	Y_12_
Donje Selo, Montenegro	R220	“thomasi”	44	44	male	X_8_	Y_6_
*M. atticus*							
Afidnes, Greece	R191	“atticus”	44	46	male	X_13_	Y_10_
Chalkida, Greece	R294	“Evia”	44	44	male	X_3_	Y_1_
Kimassi, Greece	R171	“Evia”	44	44	male	X_3_	Y_11_

^a^ Chromosomal races according to Rovastos et al. [23]. ^b^ Sex chromosome variants based on their painting profiles according to Rovastos et al. [23]. * R32 individual had two 2X_13_ and one Y_1_, according to Rovatsos et al. [44].
